# Supplementary material for: Efficient Generation of Multiple Seamless Point Mutations Conferring Triazole Resistance in Aspergillus fumigatus
Source: J Fungi (Basel). 2023 Jun 2;9(6):644. doi: 10.3390/jof9060644 (PMC10301212; doi:10.3390/jof9060644)
Supplement: Supplementary file 1 [file jof-09-00644-s001.zip › jof-2401262-supplementary.pdf]

Supplementary Tables.

Table S1. Strains used in this study.

Strain	Genotype	Source
AkuB^{KU80}	*KU80Δ*	Da Silva Ferreira et al., 2006 [8]
**Cyp51A G448S**	*KU80Δ*; *cyp51A*/G448S	This study
**Cyp51B G457S**	*KU80Δ*; *cyp51*B/G457S	This study
**Hmg1 F262del**	*KU80Δ*; *hmg1*/F262del	This study
**Cyp51B G457S /Hmg1 F262del**	*KU80Δ*; *cyp51*B/G457S; *hmg1*/F262del	This study
**Cyp51B G457S/ Hmg1 F262del/ Cyp51A G448S**	*KU80Δ*; *cyp51*B/G457S; *hmg1*/F262del; *cyp51A*/G448S	This study

Table S2. Gene amplification primers used in this study

Target gene	Primer name	Primer sequence (5' → 3')	Final construct
cyp51A	Cyp51A F	GGGCTGGAGATACTATGGCTTTCA	Mutated Cyp51A
	Cyp51A R	CAGGTTTTCGCACGAGCTTC	G448S
cyp51B	Cyp51B F	ATGGGTCTCATCGCGTTCATT	Mutated Cyp51B
	Cyp51B R	TCAGGCTTTGGTAGCGGACTC	G457S
hmg1	Hmg1 F	CAGCATCGAGTCGAGAGAATTT	Mutated Hmg1
	Hmg1 R	CTGCGTTACTCGGTCTTGGTAC	F262del
	Hmg1 OF	TATGCTGCCATATTTGCTGATG	Hmg1 F262del
	Hmg1 OR	ACGAGACAGTAGAGGTAGGCC	mutation area

Table S3. crRNAs used in this study

Target gene	5' crRNA	3' crRNA
cyp51A	GGTGCCGATGCTATGGCTTA	GAAGCCAAGCATCATCGGCT
cyp51B	ACATGGGTGCTTGTTGGAAT	AAAAGATCGGCCAAGCGGTT
hmg1	GCACCCTATACACACCATTG	GCATGGCGAAACATGAAGTA

Table S4. ARMP-PCR primers used in this study

Target gene	Primer name	Primer sequence (5' → 3')
cyp51A	Cyp51A OF	TGCTGAGACTGGCCTCACAGC
	Cyp51A WT	CACGTCAAGTCCCTATCTTCCGTGTG
	Cyp51A Mut	ATACAGCGGTGTCGGCCAGCCCT
	Cyp51A OR	GCCCTCGAGGGGCTGAATTAAGTAT
cyp51B	Cyp51B OF	CGTGTTCTCGGATCTGACTTG
	Cyp51B WT	CAATGCATCGGTGTCTACCTGATCC
	Cyp51B Mut	CCAATAGCCCGTACCTCCCGTGTA
	Cyp51B OR	TCAGGCTTTGGTAGCGGACTC

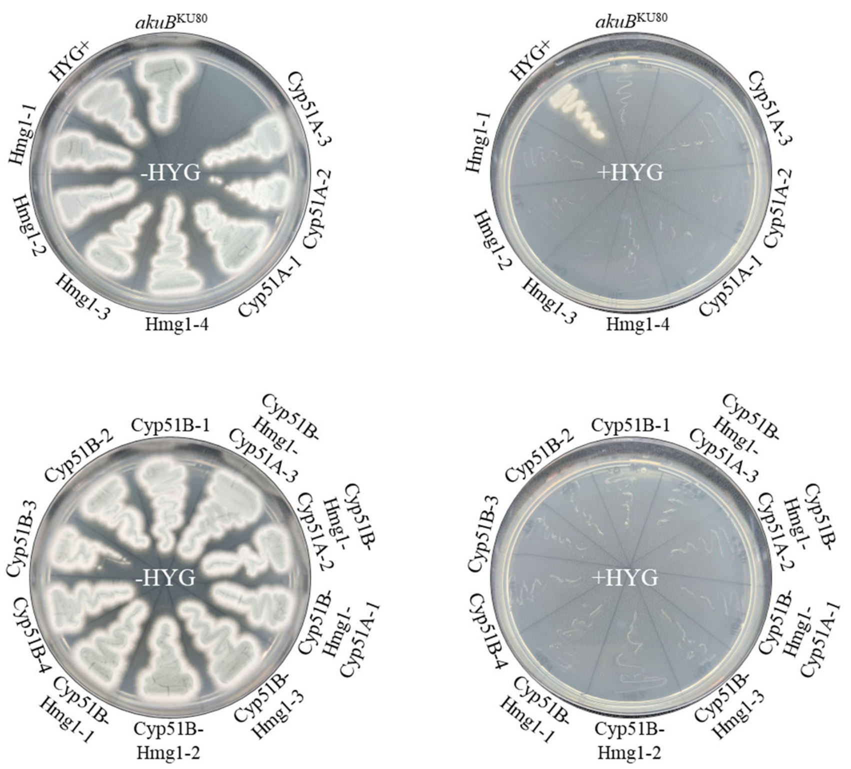

Figure S1. **Analysis of mutants for loss of hygromycin resistance**. Analysis of mutants Hmg1 F262del (Hmg1-1-4), Cyp51A G448S (Cyp51A1-3), Cyp51B G457S (Cyp51B1-4), Cyp51B G457S /Hmg1 F262del 1-3, and Cyp51B G457S/ Hmg1 F262del/ Cyp51A G448S 1-3 for loss of pTel-hyg^{R} and HYG resistance. Transformants were passaged twice on YAG absent hygromycin. Single isolates were then streaked on plates supplemented with (+HYG) or without (-HYG) hygromycin (350 µg/ml). Plates were photographed after 24 h incubation at 37C. Controls include parental strain *AkuB*^{KU80} and a positive control strain containing the hygromycin resistance cassette stably integrated into the genome (strain designated HYG+).

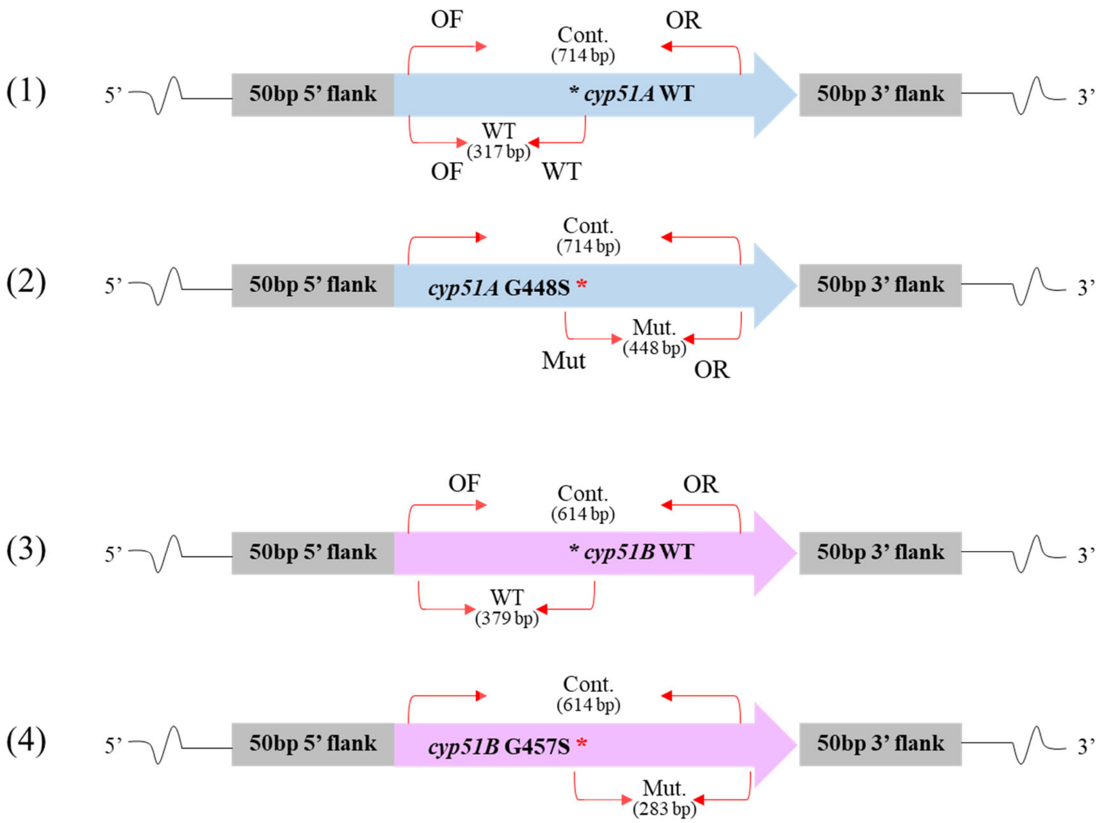

Figure S2. ARMS-PCR analysis of *cyp51A* and *cyp51B* point mutations. Cyp51A G448S detection uses three primer sets: Cyp51A OF and Cyp51A OR control primer set (S1.1), amplifying the 714 bp around the mutation (cont), Cyp51A OF and Cyp51A WT amplifying the 317 bp WT sequence if present (S1.1), and Cyp51A OR and Cyp51A Mut primer set, amplifying the 448 bp mutated (Mut.) sequence, if present (S1.2).

Cyp51B G457S detection uses three primer sets: Cyp51B OF and Cyp51B OR control primer set (S1.3), amplifying the 614 bp around the mutation (cont), Cyp51B OF and Cyp51B WT amplifying the 379 bp WT sequence if present (S1.3), and Cyp51B OR and Cyp51B Mut primer set, amplifying the 283 bp mutated (Mut.) sequence, if present (S1.4).
